# Periodic Metallo-Dielectric Structures: Electromagnetic Absorption and its Related Developed Temperatures

**DOI:** 10.3390/ma12132108

**Published:** 2019-06-30

**Authors:** Jean Paul Walker, Venkataraman Swaminathan, Aisha S. Haynes, Haim Grebel

**Affiliations:** 1 Electronic Imaging Center and Electrical Engineering Department, New Jersey Institute of Technology, Newark, NJ 07102, USA; 2 U.S. Army Combat Capabilities Development Command Armaments Center, Picatinny, NJ 07806, USA

**Keywords:** metallo-dielectric structures, electromagnetic filters, electromagnetic absorption, radiation trapping, temperature rise

## Abstract

Multi-layer, metallo-dielectric structures (screens) have long been employed as electromagnetic band filters, either in transmission or in reflection modes. Here we study the radiation energy not transmitted or reflected by these structures (trapped radiation, which is denoted—absorption). The trapped radiation leads to hot surfaces. In these bi-layer screens, the top (front) screen is made of metallic hole-array and the bottom (back) screen is made of metallic disk-array. The gap between them is filled with an array of dielectric spheres. The spheres are embedded in a dielectric host material, which is made of either a heat-insulating (air, polyimide) or heat-conducting (MgO) layer. Electromagnetic intensity trapping of 97% is obtained when a 0.15 micron gap is filled with MgO and Si spheres, which are treated as pure dielectrics (namely, with no added absorption loss). Envisioned applications are anti-fogging surfaces, electromagnetic shields, and energy harvesting structures.

## 1. Introduction

**Figure 1:**
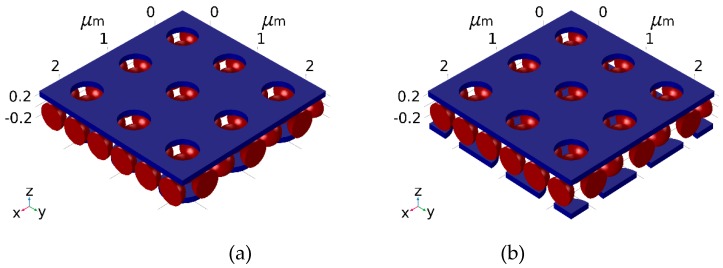
Cross section of a bi-layer metallo-dielectric screen. The top (front) metal screen is made of a hole-array; the bottom (back) screen is made of metallic disks and the gap is filled with dielectric-embedded silica colloids. (**a**) Un-shifted construction: The disk-array is centered below the hole-array. (**b**) Shifted structures: The disk array is diagonally shifted along the x-y direction by half a pitch with respect to the hole-array.

We focus on two screen structures, depicted in Figure 1. They are composed of a top (front) metal film with a hole-array. This type of screen is known as an inductive screen; on its own, the screen serves as a transmission band filter. The inductive screen is placed above an array of metallic disks. A disk-array screen is known as a capacitive screen; on its own it serves as a reflection band filter. Both metal screens are separated by a dielectric layer, which is embedded with dielectric colloids. Suspended monolayer or multilayers of individual screens have been used as electromagnetic bandpass filters in the mid- to far-infrared (IR) [1,2,3,4] and in the visible and near-IR [5,6,7], albeit without the separating colloidal layer. They are typically employed in the long wavelength regime where the screen’s pitch is smaller than the incident wavelength. The screens operate at resonance; local modes in the screen’s opening [8,9] are coupled with extended modes that propagate along the metal surface [10,11,12,13]. One speaks of surface plasmon polariton (SPP) modes when a complex refractive index is used [14], and of surface waves when the boundary conditions for the surface impedance are applied [15]. Metal screens play an important role in infrared astronomy [16] and remote sensing. Free standing metal screens are commercially available and have been used as band pass filters for several applications [1,17,18]. The filters isolate desired infrared signals from more energetic short wavelength radiation, allow temperature measurements, provide order sorting for grating spectrometers, and improve signal-to-noise ratio for Fourier Transform (FT) spectrometers. The concept has been extended to curved screens [19,20] and was applied to sensing and diagnostics as well [21,22,23]. Here we assess the electromagnetic energy, captured between a bilayer structure that is a combination of inductive and capacitive screens and whose gap is filled with closed-packed colloids. We study the effect of the captured energy on the developed temperatures in the absence of a heat sink.

**Figure 2:**
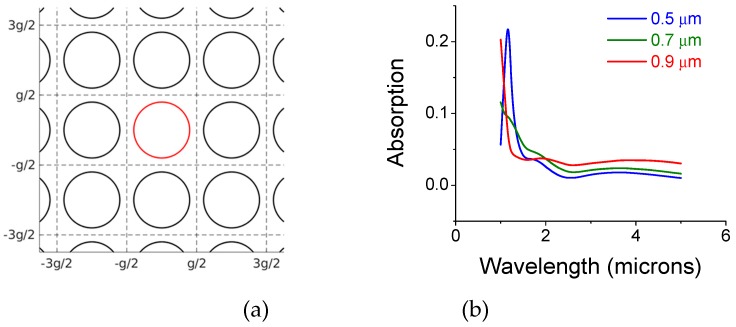
Monolayer copper screen with opening in air. (**a**) The screen structure; (**b**) Intensity absorption coefficient as a function of wavelength for various hole-diameters: 0.5 microns (blue curve); 0.7 microns (green curve); 0.9 microns (red curve). The pitch, **g**, is g_x_ = g_y_ = 1 microns. The copper thickness is 0.1 microns.

In Figure 2a we show a standalone single, 0.1 micron thick copper film with circular openings in air (an inductive screen). The screen transmits a certain band of wavelengths, which depends on the screen periodicity, thickness, and opening. The transmission is almost eliminated when the opening is 10% of the 1-micron pitch. The intensity not reflected or transmitted is denoted absorption and is plotted in Figure 2b. Screens with relatively small holes tend to reflect most of the radiation intensity back; therefore, less power is propagating along the screen surface. 

As mentioned earlier, individual screens may be characterized as bandpass filters either in transmission or in reflection mode of operation. Less prevalent is their absorption [24,25] or their related heat mode of operation. Their transmittance (or reflectance) depends on the metal film thickness, hole or patch dimension and shape, angle of incidence and their complex permittivity values (that is, the interplay between the negative dielectric constant and the metal conductivity). The filters operate in the long wavelength regime where the screen pitch is equal to, or smaller than the incident wavelength. Therefore, it should come as no surprise that the screen opening is smaller than the radiation wavelength. The incident radiation is coupled to a surface mode via momentum conservation with the periodic structure (see below). The surface wave is then coupled to the back side of the front screen via aperture modes. These aperture modes may be either propagating along the aperture axis, or may be evanescent. For gaps smaller than the incident wavelength, the radiation is directly coupled to the front surface of the second (back) screen and finally finds itself at the structure’s output as a free-space mode. Intuitively, the aperture may be considered as a waveguide across the thickness of the metal film. Very small apertures operate below a waveguide cut-off; the coupling between the front and back screens are made through evanescent modes. On the other hand, large screen openings—larger than half the incident wavelength lead to diffraction. 

Dispersion relations dictate the propagation constant of the surface waves. For example, the surface propagation constant along the x-direction, β_x_ is related to the pitch along the x-direction, g_x_ and the propagation constant of the incident beam k_0_ as: β_x_ = k_0_sin(θ) ± q2π/g_x_, with q—an integer. Resonance occurs whenever the propagating surface wave becomes a standing wave. For example, at normal incidence (θ = 0) the surface mode is made of two counter propagating modes, β_x_ = ±q2π/g_x_, and the total momentum is equal to zero. For oblique incidence, θ ≠ 0, there are four surface wave components, two on either side of each metal screen. Thus, the overall efficiency and the bandwidth of the filter is dictated by the pitch, the opening size, and the metal film thickness [7,26,27,28]. 

## 2. Simulations

The simulations used a finite element code (MultiPhysics, Comsol) on a cell size of 1 × 1 × 10 micron^3^. The linearly polarized plane wave along the y-direction propagates along the (negative) z-direction and has an incident intensity of 1 W. The fluence of radiation is, therefore, 10^8^ W/cm^2^. Periodic boundary conditions are used with a perfect matched layer on the top and bottom of the cell. As for the thermal simulations, we used periodic boundary conditions around the edges of the unit cell. For example, we set the temperature along the x-direction, temperature(x,−g/2,z) = temperature(x,g/2,z), and along the y-direction, temperature(−g/2,y,z) = temperature(g/2,y,z)). We used insulation boundary conditions on any surface that has a solid–air boundary inside the unit cell meaning that there is no heat transfer normal to the surface boundary. The simulation works in one direction: The electromagnetic simulator affects the currents on the conductive layers, which result in heat. One may go one step further to assess how the heat affects the electromagnetic absorption (via the thermal expansion coefficient and the change in the metal impedance). This step is briefly described at the end of this paper and will be part of future works. The full-wave finite element solver with appropriate boundary conditions provides a complete solution to electromagnetic problems and found in line with previous experimental results [19]. 

**Figure 3:**
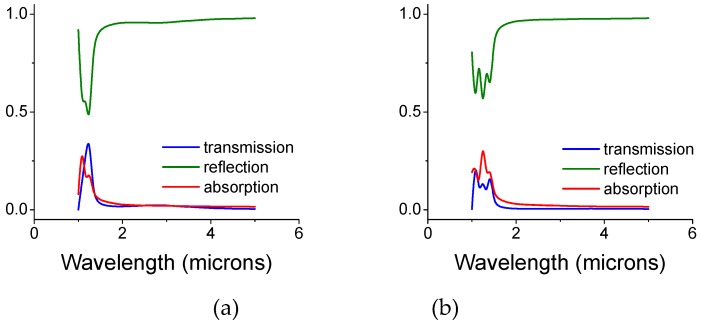

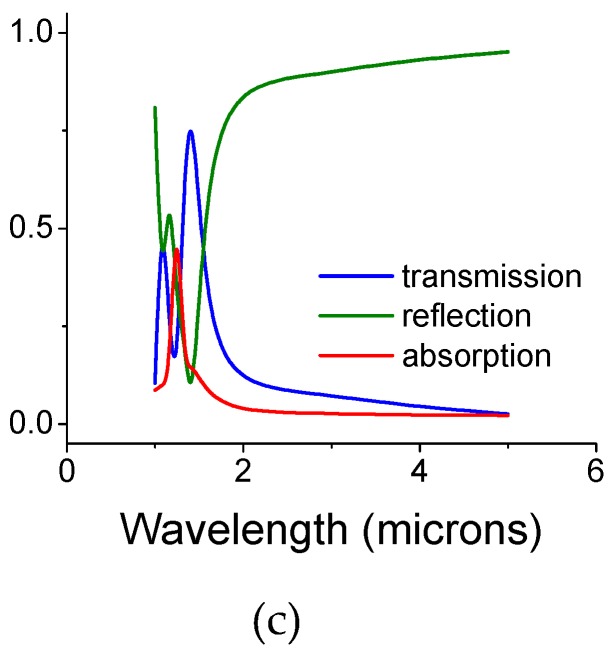
Intensity coefficients, transmission, reflection, and absorption for an un-shifted bi-layer screen whose air gap is filled with silica colloids. The opening and disk diameters are 0.5 and 0.7 microns, respectively. (**a**) The gap is 0.25 microns. (**b**) The gap is 0.5 microns. (**c**) The opening and disk diameters are interchanged: 0.7 and 0.5 microns, respectively, for a gap of 0.5 microns. The thickness of each copper mono-screen is 0.1 microns and the diameter of the colloids equals the gap size.

In Figure 3, we present the intensity coefficients for the transmission, reflection, and absorption (defined here as A = 1 − T − R) for a bi-layer screen whose air gap size is 0.25 and 0.5 microns. The gap is filled with silica colloids whose diameter is the same as the gap and may be viewed as convenient spacers. The diameter of the front openings is d_open_ = 0.5, while the back disk diameter is d_disk_ = 0.7 microns, respectively. For thin metal screens, the key parameters are the gap size (see below) and the screen periodicity. 

**Figure 4:**
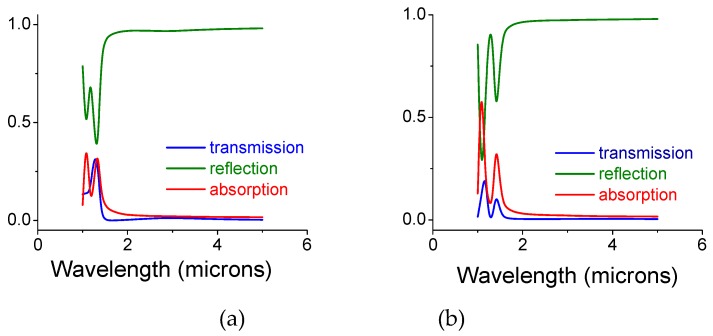
Intensity coefficients, transmission, reflection, and absorption for a diagonally shifted bi-layer screen whose air gap is filled with silica colloids in air. The opening and disk diameters are 0.5 and 0.7 microns, respectively. (**a**) The gap is 0.25 microns. (**b**) The gap is 0.5 microns. The thickness of each copper mono-screen is 0.1 microns and the diameter of the colloids equals the gap size.

In Figure 4 we show a similar situation; however, for a diagonally shifted bi-layer structure (Figure 1b). The diameter of the front openings and the back disks remained, d_open_ = 0.5 and d_disk_ = 0.7 microns, respectively. Relatively large absorption at 1.1 microns is noted for a gap of 0.5 microns (Figure 4b). Overall, and upon a proper screen’s design the screens parameters may exhibit an absorption coefficient above 90% at a particular wavelength, λ (Figure 6)). 

**Figure 5:**
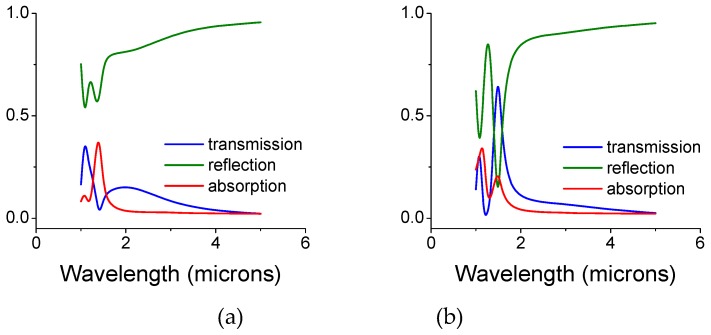
Intensity coefficients for transmission, reflection, and absorption for a diagonally shifted bi-layer screen whose air gap is filled with silica colloids in air. The opening and disk diameters are the reverse of Figure 3: 0.7 and 0.5 microns, respectively. (**a**) The gap is 0.25 microns. (**b**) The gap is 0.5 microns. The thickness of each copper mono-screen is 0.1 microns and the diameter of the colloids equals the gap size.

In Figure 5 we show a diagonally shifted bi-layer structure (Figure 1b), while interchanging the diameters of the front openings and the back disks: d_open_ = 0.7 and d_disk_ = 0.5 microns, respectively. Large peak transmission at a gap of 0.5 microns, is noted. 

**Figure 6:**
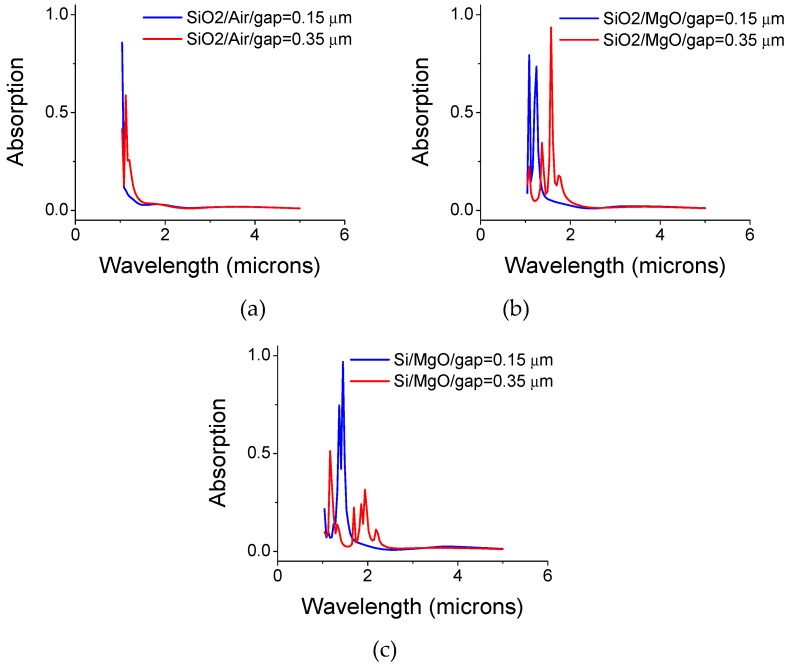
(**a**) Silica sphere in air filled gap, and (**b**) silica spheres embedded in MgO filled gap for two gap values: 0.15 and 0.35 microns. (**c**) Si spheres embedded in MgO filled gap.

The gap and its material composition are major parameters to be considered (Figure 6). As an example, large absorption peaks may be observed for gap values of 0.15 and 0.35 microns. As shown in Figure 6b, one achieves 93% intensity absorption at λ = 1.58 microns with a hole-diameter of 0.5 microns, disk diameter of 0.5 microns, and a gap of 0.35 microns for a host MgO, embedded with silica spheres. The spheres diameter is 0.35 microns similar to the gap size. Note that the refractive index of the silica spheres (n = 1.45) is smaller than the surrounding host MgO material (n = 1.7). When replacing the silica spheres with silicon (n = 3.5, k = 0) an absorption of 97% is exhibited at λ = 1.45 microns and a gap of 0.15 microns (Figure 6c). Note the peak shift for the larger gap, as well as when replacing air with MgO. 

**Figure 7:**
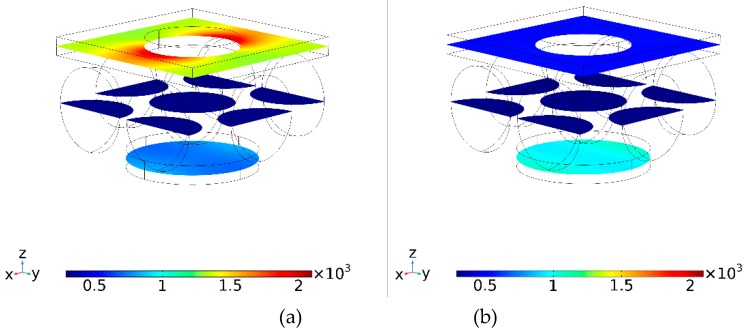
Temperature distribution in Kelvins (^o^K) after 1 ns at peak electromagnetic absorption wavelength of (**a**) λ = 1.24 microns and (**b**) λ = 1.33 microns when the gap is filled with air/silica spheres.

The effect of heat is shown in Figure 7 for an un-shifted bi-layer screen with air-embedded silica spheres. Figure 7a is for λ = 1.24 microns (the first absorption peak in Figure 3b) while Figure 7b is for λ = 1.33 microns (the second absorption peak in Figure 3b). The structure’s dimensions were as follows: pitch—1 microns; front aperture diameter: 0.5 micron; gap: 0.5 microns; sphere diameter: 0.5 microns and back disk diameter: 0.7 microns. The incident radiation is 1 W per 1 × 1 micron^2^ cell. Note the switch the roles of colder and hotter screen when the wavelength is changed by merely 0.09 microns. This is due to variation in radiation flow from the front to the back screen as a function of wavelength.

**Figure 8:**
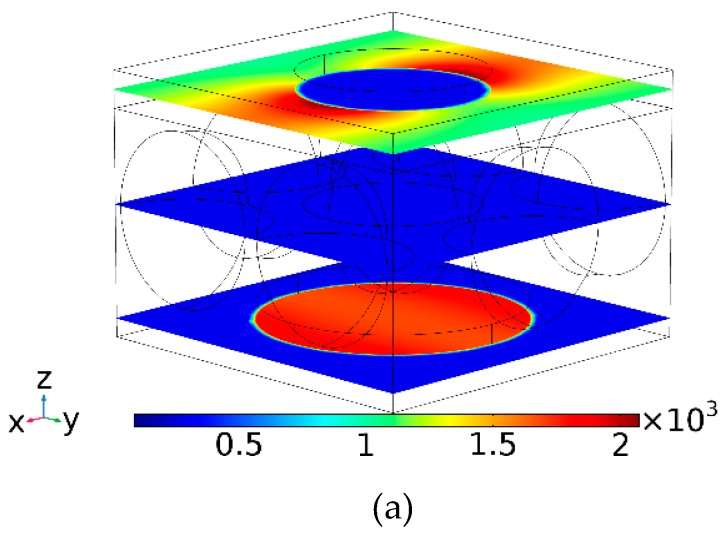

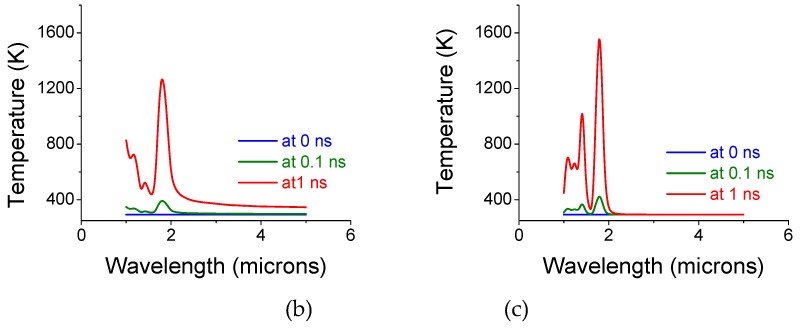
(**a**) Temperature distribution in Kelvins (^o^K) after 1 ns at peak electromagnetic absorption wavelength (λ ~1.8 microns) when the gap is filled with polyimide/silica spheres (n_polyimide_ = 1.7/n_silica_ = 1.45). (**b**) Average temperature at the top screen as a function of wavelength at 0, 0.1, and 1 ns of exposure. (**c**) Average temperature at the bottom (back) copper disks.

A similar structure albeit with polyimide/silica spheres (n_polyimide_ = 1.7/ n_silica_ = 1.45) in its gap is shown in Figure 8a. Figure 8b,c each show three curves corresponding to average temperatures at 0, 0.1, and 1 ns for the polyimide filled sample. The temperature of the gap volume remains at room temperature. For polyimide, the back disk becomes hotter. 

**Figure 9:**
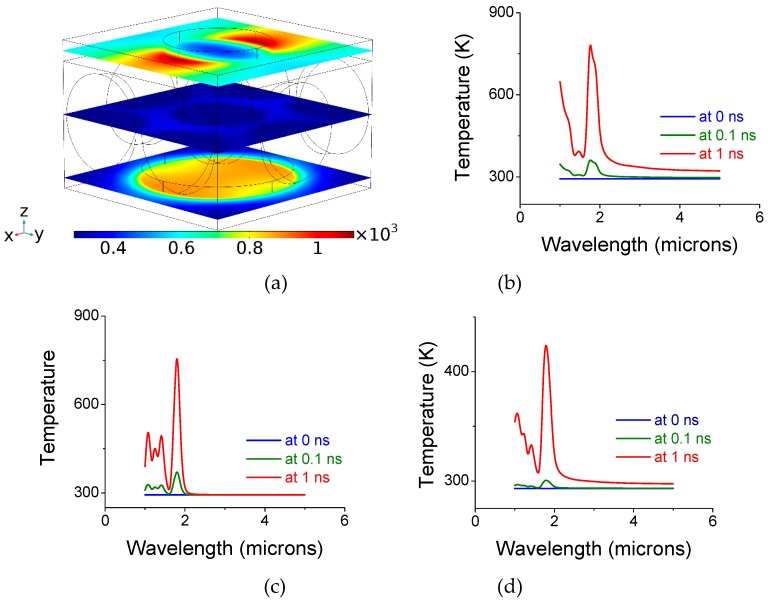
Temperature distribution and absorption for un-shifted bi-layer screen. (**a**) Temperature distribution in Kelvins (^o^K) after 1 ns at peak electromagnetic absorption wavelength (λ ~1.8 microns). (**b**) Average temperature at the top screen as a function of wavelength at 0, 0.1, and 1 ns of exposure. (**c**) Average temperature at the bottom (back) copper disks. (**d**) Average temperature of the colloids. The bi-layer structure was embedded with silica colloids in MgO.

Air and polyimides are known to be poor heat conductors. In Figure 9, we show results for an un-shifted bi-layer screen. Its gap was filled with magnesium oxide (MgO), embedded with silica colloids. MgO is a good heat conductor with refractive index similar to polyimide, n~1.7. The dimensions for the top opening, disk diameter, and gap/sphere-diameter, are respectively: 0.5, 07, and 0.5 microns. The incident radiation is 1 W per 1 × 1 micron^2^ surface. We show three curves corresponding (from bottom to top) to average temperatures at 0, 0.1, and 1 ns. As can be seen from Figure 9, the overall temperature is lower when compared with Figure 8. The temperature of the gap volume has increased due to the heat conductive MgO layer. The main accentuated peak of Figure 9 has shifted to longer wavelengths when compared with Figure 3. The resonance wavelengths are red-shifted when the screen is filled with a larger refractive index. 

**Figure 10:**
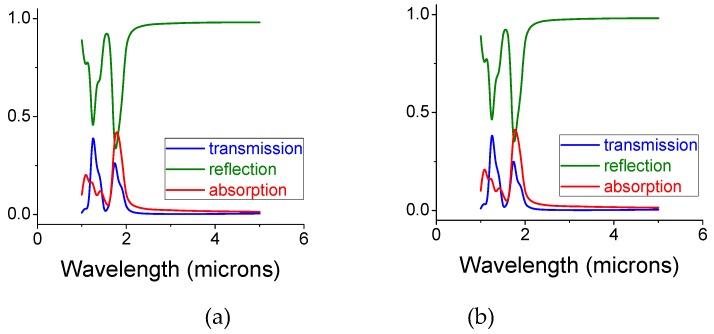
Intensity coefficients for transmission, reflection, and absorption for an un-shifted bi-layer screen filled with silica colloids in MgO. The structure was held at (**a**) room temperature and (**b**) at 850 °C. The opening and disk diameters are 0.5 and 0.7 microns, respectively, and the gap size and the sphere diameter are 0.5 microns. The thickness of each copper mono-screen is 0.1 microns and the diameter of the colloids equals the gap size. No significant change is noted in the electromagnetic coefficients for the ‘cold’ and ‘hot’ cases.

So far, we have used one-step simulations where the electric field resulted in induced currents and hence heat. However, one may ask: What would happen if the elevated temperature affects the optical properties of the screen while heating up? Such self-consistent analysis is possible but requires a much powerful computer than we have. Instead, we analyzed the same model, subjected to heat at 850 °C while using well-tabulated heat expansion coefficients for each material component. The results are shown in Figure 10a,b. When comparing Figure 10a to Figure 10b, we note that the major absorption wavelength—the wavelength at ca 1.8 microns that results in substantial heat—only slightly changes. This is because the gap size and the periodicity did not change much as a function of temperature. The gap is filled with silica colloids embedded in MgO, which maintained their dimensions even at these large temperatures. Copper is more prone to thermal changes; however, since the simulations are conducted for an infinite screen (through periodic boundary conditions) the screen thickness is the parameter to be mostly affected. However, the screen thickness only weakly affects the resonance wavelength. Thus, when comparing the electromagnetic coefficients of Figure 10a and Figure 10b, there is only slight change between the ‘cold’ (room temperature) and ‘hot’ (850 °C) cases. 

How would one implement these structures? Silica colloids are rather easy to make with precise diameters. An ordered array of silica colloids, known as opal, are fabricated through deposition of successive monolayers by using a dipping technique. Its refractive index may be modified through ion implantation [29,30]. The coating may be made on dielectric or on lithographically patterned metallic substrates. The voids in the silica colloidal array may be filled with another dielectric component such as polyimide, or a glassy material such as MgO. Furthermore, the original silica array may be etched away and be replaced by another component [31], e.g., amorphous silicon. The top metal screen may be then be lithographically defined. 

## 3. Conclusions

Electromagnetic radiation, trapped within bi-layer screens results in a rapid temperature increase. We have analyzed the temperature distribution over time for various incident wavelengths when the structures are decoupled from a heat sink. One may achieve an electromagnetic intensity absorption of 97% when the bi-layer screen’s gap is filled with Si spheres, embedded in MgO. Optimizing the screen parameters (lattice structure, lattice constants, aperture and disk diameters, metal film thickness, and gap filling materials) may enable applications, such as energy harvesting, electromagnetic shielding, and anti-fogging surfaces. Future works could improve on the absorption parameters to make it broadband [25].
